# High-Energy Milling as a Pre-Treatment Alternative for Lignocellulosic Fibers Derived from Brewer’s Spent Grain

**DOI:** 10.3390/polym17091156

**Published:** 2025-04-24

**Authors:** Erik Gomez-Hernandez, Ernesto Hernández-Hernández, Javier Castro-Rosas, Rosa A. Vázquez-García, Arturo Cadena-Ramírez, Brenda E. Jiménez-Villeda, Carlos A. Gomez-Aldapa

**Affiliations:** 1 Doctorado en Ciencias Ambientales, Área Académica de Química-ICBI, Ciudad del Conocimiento, Carretera Pachuca-Tulancingo Km. 4.5, Colonia Carboneras, Mineral de la Reforma 42184, Hidalgo, Mexico; go111626@uaeh.edu.mx; 2 Departamento de Materiales Avanzados, Centro de Investigación en Química Aplicada (CIQA), Blvd. Ing. Enrique Reyna H. No. 140, Col. San José de los Cerritos, Saltillo 25294, Coahuila, Mexico; ehernandez@ciqa.edu.mx; 3 Área Académica de Química-ICBI, Ciudad del Conocimiento, Carretera Pachuca-Tulancingo Km. 4.5, Colonia Carboneras, Mineral de la Reforma 42184, Hidalgo, Mexico; jcastro@uaeh.edu.mx; 4 Área Académica de Ciencias de la Tierra y Materiales-ICBI, Ciudad del Conocimiento, Carretera Pachuca-Tulancingo Km. 4.5, Colonia Carboneras, Mineral de la Reforma 42184, Hidalgo, Mexico; 5 Programa de Ingeniería Biomedica, Universidad Politécnica de Pachuca, Carretera Pachuca-Cd. Sahagún Km. 20 Ex-Hacienda de Santa Bárbara, Zempoala 43830, Hidalgo, Mexico; arturocadena@upp.edu.mx; 6 Tecnológico Nacional de México Campus Occidente, Paseo del Agrarismo 2000, Car. Mixquiahuala-Tula, Km. 2.5, Mixquiahuala de Juárez 42700, Hidalgo, Mexico; bjimenez@itsoeh.edu.mx

**Keywords:** agro-industrial residue, brewery spent grain, high-energy ball milling, lignocellulosic fiber, pretreatment

## Abstract

The objective of this study was to evaluate how high-energy milling affects the structural, thermal, and morphological properties of brewer’s spent grain fibers over time. The researchers determined the chemical composition of the samples using TAPPI techniques, particle size analysis, Fourier-transform infrared spectroscopy (FTIR), scanning electron microscopy (SEM), thermogravimetric analysis (TGA), and X-ray diffraction (XRD). The samples displayed distinct morphologies and particle sizes depending on the treatment duration. The sample treated for 120 min (T120) showed the smallest particle size (19.4 µm). FTIR spectra revealed that the mechanical treatment strongly disrupted the structure of hemicellulose. The thermal stability of the samples decreased because of the applied treatment. Mechanical milling also fully eliminated the crystalline structure of cellulose in the samples. These findings indicate that high-energy milling holds strong potential as a pre-treatment method for the valorization of lignocellulosic residues.

## 1. Introduction

Brewer’s spent grain (BSG) represents the most abundant lignocellulosic waste in the brewing industry, accounting for 85% of the total waste produced. Breweries generate approximately 6.2 kg of BSG per hectoliter of beer [1]. In 2018, global production of BSG reached 38.8 million tons [2]. Currently, 70% of BSG serves as feed for cattle, poultry, and chickens; 10% supports gas production; and the remaining 20% ends up in landfills, releasing millions of tons of CO_2_ greenhouse gases [3]. Researchers have identified cellulose, hemicellulose, lignin, lipids, and a small protein fraction as BSG’s main components. Its composition depends on both raw materials and processing steps.

Thanks to its chemical composition, abundance, and low cost, BSG offers strong potential as a raw material for various commercial processes. It serves as a source of bioactive compounds for human nutrition, a food additive, and a substrate for enzyme production [4]. BSG also finds use as an absorbent material, and in producing biogas [5,6], hydrogen, and biofuels [7].

The effective valorization of BSG requires techniques that disrupt the crystalline organization of cellulose, fragment lignin, and hemicellulose. In this context, pre-treatment methods play a key role by enabling the exposure of different components for efficient use [8]. Ball milling has emerged as a promising approach and currently draws significant interest from researchers. This physical treatment applies mechanical energy to reduce particle size, crystallinity, and the degree of polymerization of lignocellulosic materials without producing toxic wastes [9]. Ball milling not only pulverizes materials mechanically but also triggers mechanochemical effects by depolymerizing macromolecules in the cell wall. Research has shown that cellulose, hemicellulose, and lignin form extensive molecular and macromolecular networks. These networks interact with each other and limit both the accessibility and digestibility of lignocellulosic biomass.

Liu et al. [10] reported that ball milling effectively reduces the particle size of BSG and causes a significant decrease in the crystallinity of cellulose. They attributed this reduction in crystalline to both the fragmentation and mechanical deformation of cellulose fibers. Their experimental results were conclusive and demonstrated that smaller particle size and reduced crystallinity promote the bioconversion of cellulose, which in turn increases the total production of volatile fatty acids (VFA) from 0.25 to 0.33 g/g VS. Additionally, another research group observed that the strong mechanical action applied during milling for 120 min not only altered the particle size but also caused severe damage to the crystalline structure of cellulose. The crystallinity index decreased from 93% to 51%, thus facilitating enzymatic hydrolysis and enhancing the conversion of cellulose into fermentable sugars [11]. Zhang et al. [12] also demonstrated significant changes in the microstructure of corn stover subjected to ball milling. Lignin was substantially disrupted, while cellulose and hemicellulose broke into smaller fibers. This mechanical treatment also increased the surface area and porosity, thereby improving cellulose accessibility and enzymatic hydrolysis efficiency in hydrogen production. Ji et al. [13] showed similar results using rice straw, reporting enhanced glucose yields after mechanical pretreatment. These findings support high-energy ball milling as an efficient method for large-scale biomass valorization [14,15].

Although numerous investigations have examined brewer’s spent grain (BSG) and several of its pretreatment strategies, researchers have not yet fully documented how ball milling affects BSG’s structural and compositional characteristics. This study aimed to evaluate the effect of high-energy milling, over time, on the chemical, structural, thermal, and morphological properties of lignocellulosic fibers derived from BSG.

### General Workflow

**Figure 1:**
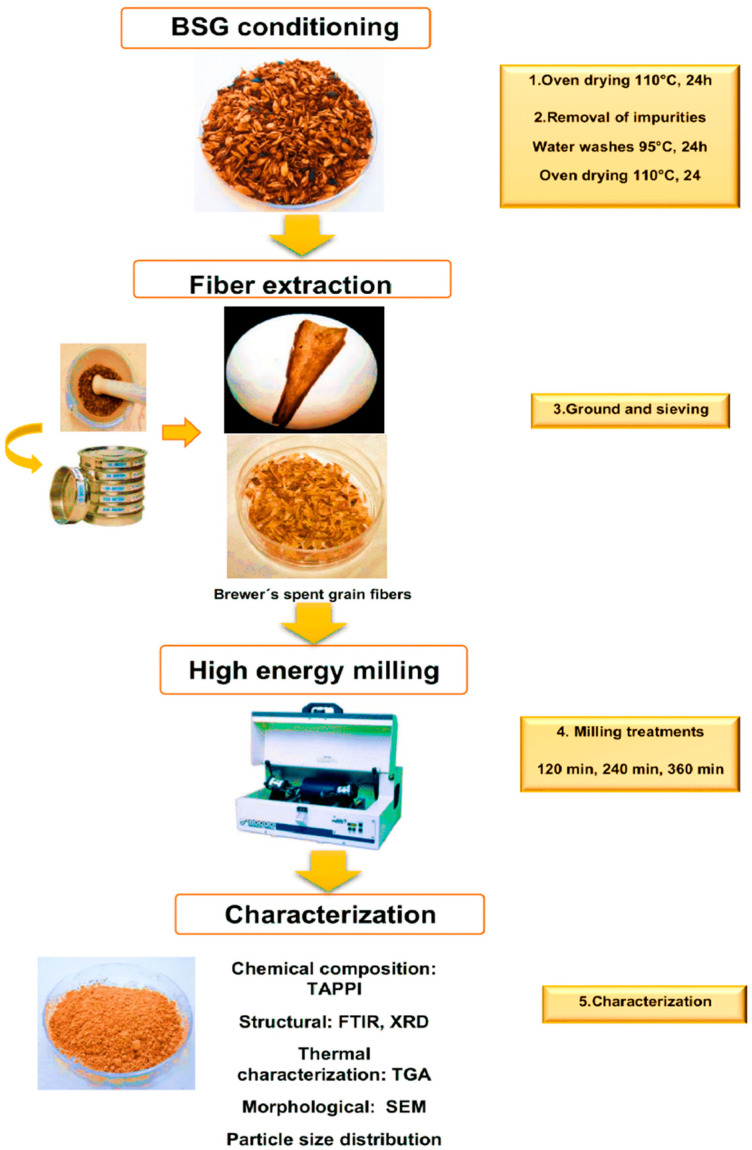
General workflow of the experimental procedure for high-energy milling of brewer’s spent grain fibers and the respective characterization techniques.

The researchers applied the following methodological framework to extract and characterize brewery spent grain fibers (Figure 1).

## 2. Materials and Methods

The microenterprise La Hacienda, located in the municipality of Zempoala, Hidalgo, Mexico, provided the brewer’s spent grain (BSG). The researchers obtained the sample directly from a production batch and applied the quartering method for collection. To prevent decomposition, they removed excess moisture by placing the sample in a drying oven (Thermolyne, Oven Series 9000, Waltham, MA, USA) at 110 °C for 24 h. They then placed 500 g of BSG in a pot with 5 L of water and heated the mixture to 95 °C for 1 h with manual stirring. Afterward, they filtered the mixture and repeated the procedure five times to remove yeast residues, endosperm fragments, and water-soluble components. The researchers dried the resulting material in the same oven (Thermolyne, Oven Series 9000, Waltham, MA, USA) at 110 °C for another 24 h. This process ensured proper sample preparation for subsequent analysis and characterization of the BSG fibers under controlled and reproducible conditions.

### 2.1. Extraction of Fibers from Brewer’s Spent Grain

The researchers applied a combination of physical methods to remove endosperm residues from the barley grains. They ground the dried BSG in a porcelain mortar for 3 min and sieved it through mesh numbers 10, 12, and 14 (USA Number), obtaining fibers between 750 µm and 2 mm. They stored the resulting BSG fibers in plastic bags and performed chemical, structural, and thermal characterization.

### 2.2. Effect of Milling Conditions on Composition Particle Size Distribution, and Morphology of Lignocellulosic Material

The researchers studied the effect of milling time on particle size. They used zirconium oxide balls with a diameter of 3.5 mm at three different milling durations (120, 240, and 360 min), later labeled as T120, T240, and T360. For each run, they placed 1 g of BSGFs into a stainless-steel container along with 14 g of zirconium oxide balls (3.5 mm). They performed the milling process using a high-energy mill (Spex, Model 800D, Metuchen, NJ, USA). After milling, they stored the samples in glass containers for further characterization.

### 2.3. Chemical Composition

The researchers determined the percentage of soluble extractable compounds using TAPPI 204 cm-97. They measured the cellulose percentage in both BSGFs and the resulting samples following TAPPI-203 os-74, as established by the Technical Association for the Pulp and Paper Industries. They quantified lignin content according to TAPPI 22 om-98. For cellulose content, they applied the modified Kurschner and Hoffer method from TAPPI (1978), while they calculated the holocellulose content using the method by Wise et al. [16]. All measurements were performed in triplicate.

#### 2.3.1. Determination of Soluble Extractible Compounds

The researchers applied the TAPPI 204 cm-97 technique to determine the soluble extractable compounds. They placed a 5 g sample in an extraction cartridge and positioned it inside a Soxhlet extraction apparatus. The procedure included three solvents used in successive stages: an ethanol-toluene mixture (1 part ethanol to 2 parts toluene), 96% ethanol, and hot water. They maintained the apparatus in recirculation for 4 h with each solvent, aiming for an average of four cycles per hour. After completing the extraction, they removed the cartridge and placed it in an oven (Thermolyne, Oven Series 9000, Waltham, MA, USA) at 80 °C for 2 h.

The percentage of extractives was calculated using the following formula:

$$\%\;\mathrm{Soluble}\;\mathrm{extractible}\;\mathrm{compounds}=\frac{Weight\;of\;residue\;(g)}{Weight\;of\;sample\;(g)}\times 100$$

#### 2.3.2. Determination of Lignin

The procedure followed the TAPPI 222 om-98 standard. A 1 g portion of extractive-free sample was placed in a 100 mL beaker, and 15 mL of 72% sulfuric acid was added slowly. The beaker was covered with a watch glass and kept for 2 h, with occasional stirring using a glass rod. After this period, the mixture was transferred to a 1000 mL flask, and 560 mL of distilled water was added. The solution was boiled for 4 h. After boiling, the flask was left in an inclined position overnight to allow decantation and separation of solid residues.

The solution passed through pre-weighed Whatman no. 4 filter paper. The resulting residue was washed with 100 mL of hot distilled water and dried in an oven (Thermolyne, Oven Series 9000, Waltham, MA, USA) at 105 °C for 12 h. Final weight was then recorded.

The lignin content was calculated using the following formula:

$$\mathrm{Lignin}\;\mathrm{Content}=\frac{Weight\;of\;dried\;residue\;(g)}{Weight\;of\;original\;sample\;(g)}\times 100$$

#### 2.3.3. Determination of Cellulose

Cellulose content was determined in triplicate using the modified Kurschner and Hoffer method (TAPPI, 1978). A 0.5 g portion of extractive-free sample was weighed and placed in a 100 mL beaker. Then, 2.5 mL of concentrated nitric acid and 10 mL of absolute ethanol were added. The mixture was heated in a water bath at 75 °C for 30 min. Afterward, the supernatant was decanted, and the process was repeated twice. Following the second treatment, the supernatant was removed, 4 mL of distilled water was added, and the mixture was boiled for 1 h. Centrifugation at 4000 rpm separated the solid, and the supernatant was discarded. The remaining solid was washed with 30 mL of saturated sodium acetate solution using pre-weighed filter paper and a fine-porosity Buchner funnel. Then, 200 mL of hot, distilled water was added. The filtered material was dried at 105 °C for 12 h. After drying, the sample was placed in a desiccator and weighed to determine cellulose content.

The cellulose content was calculated using the following formula:

$$\mathrm{Cellulose}\;\mathrm{Content}=\frac{Weight\;of\;dried\;residue\;(g)}{Weight\;of\;original\;sample\;(g)}\times 100$$

#### 2.3.4. Determination of Holocellulose

Holocellulose refers to the water-insoluble carbohydrate fraction found in plant materials and includes both cellulose and hemicellulose. Determination followed the method by Wise et al., 1946 [16]. A 1 g portion of extractive-free sample (P1) was placed in a 100 mL beaker. Then, 0.3 g of sodium chlorite and 32 mL of water, previously mixed with two drops of glacial acetic acid, were added. The beaker was covered with a watch glass and placed in a water bath at 75 °C for 1 h, with stirring every 30 min. After this period, two drops of acetic acid and 0.32 g of sodium chlorite were added slowly, stirred, and left to stand for 1 h. This treatment was repeated two more times, totaling 4 h. The mixture was then cooled to room temperature and vacuum filtered through pre-weighed Whatman filter paper (P2). The retained material was washed with 200 mL of hot water followed by 100 mL of acetone. Finally, the sample was dried in an oven (Thermolyne, Oven Series 9000, Waltham, MA, USA) at 40 °C for 3 days. After drying, the sample was weighed (P3).

The holocellulose content was calculated using the following formula:

$$\mathrm{Holocellulose}\;\mathrm{Content}=\frac{P3\left(g\right)-P2(g)}{P1(g)}\times 100$$

After determining the percentage of holocellulose, the hemicellulose content was calculated by subtracting the cellulose content from the holocellulose content, applying the following formula:

Hemicellulose Content=Holocelullose Content−Cellulose Content

### 2.4. Particle Size Analysis

To evaluate the effect of the factors mentioned above, a particle size analyzer (Beckman & Coulter, Model LS 1320, Brea, CA, USA) was used. This equipment determines particle size distribution in liquid suspensions based on the principle of light scattering. For the analysis, 0.25 g of sample from each milling treatment was weighed and suspended in 500 mL of distilled water. The suspension was then analyzed in the equipment to obtain the particle size distribution corresponding to each treatment.

### 2.5. Structural Characterization of Brewer’s Spent Grain Fibers (BSGFs)

#### 2.5.1. Fourier-Transform Infrared Spectroscopy (FTIR)

Structural characterization of BSGFs and the microstructures obtained after milling treatments (T120, T240, T360) was carried out using a Fourier-transform infrared (FTIR) spectrometer (Perkin Elmer, Waltham, MA, USA) within the range of 500 to 4000 cm^−1^.

#### 2.5.2. X-Ray Diffraction

X-ray diffraction analysis was performed using a Bruker diffractometer (Bruker AXS GmbH, Karlsruhe, Germany) under the following conditions: Cu-*K*α radiation source (1.5418 Å), 40 kV voltage, and 20 mA current. The crystallinity index of the fibers was calculated according to the method by Segal et al. [17]., using Origin Pro 2018 software, which allows for the separation of cellulose’s two fractions by Gaussian curve fitting for each peak, as shown in Equation (1).

(1) $$CI=\frac{I_{200}-I_{am}}{I_{200}}\times 100$$

where *I*_200_ and *I_am_* correspond to the intensity of the crystalline and amorphous peaks of cellulose present in the fiber, respectively. Scherrer’s formula, represented in Equation (2), was used to calculate the crystal size.

(2) L=(K λ)/βCos θ

where *k* = 0.89, is the Scherrer constant; β is the full width at half maximum (FWHM) of the peak; and λ is the wavelength of the radiation used.

### 2.6. Thermal Characterization

Thermogravimetric analysis was performed using a TA Instruments model Q-500 New Castle, DE, USA, within a temperature range of 30 to 600 °C, with a heating rate of 10 °C/min under a nitrogen atmosphere at a flow rate of 100 mL/min. Oxygen was introduced from 600 °C up to 700 °C to promote oxidation of the residual carbonaceous material.

### 2.7. Morphological Study

The morphology of the fibers and the microstructures obtained after milling was studied using a scanning electron microscope (JOEL JSM-7401F, Tokyo, Japan).

## 3. Results and Discussion

### 3.1. Chemical Composition of Brewer’s Spent Grain Fibers and Treatments

The chemical composition of the samples, brewer’s spent grain fibers, and the three treatments is presented in Table 1.

Brewer’s spent grain contains various grain fractions, primarily the endosperm and pericarp. Its chemical composition was 27.61% cellulose, 32.62% hemicellulose, 28.89% lignin, and 10.88% starch (calculated by difference). These values align with those reported by other authors [18]. In terms of lignin content, the value differed from that reported by Klimek [19] and closely matched the result published by Mussatto [6] (27.8%). The concentration of these constituents in BSGFs influences their mechanical and thermal behavior. These properties vary depending on factors such as barley variety, harvest season, and environmental conditions during cultivation.

Ball milling pretreatment subjects the samples to different types of mechanical stress, including shear, cutting, and compression, which induce structural changes. These changes explain the differences in cellulose, hemicellulose, and lignin percentages among T120, T240, and T360 compared to untreated BSGFs. As shown in Table 1, sample T360 exhibited the highest cellulose percentage (45.15%). This increase relates to the structural disruption caused by milling. According to Zhang et al. [12], ball milling severely destroys corncob fibers, generating porous structures that expose numerous cellulose microfibers, which lead to a higher cellulose content. In addition, T360 showed the lowest lignin content (27.86%), which reflects the mechanical depolymerization induced by milling. The variations in component contents result from mechanical stresses such as impact, compression, friction, and shear during pretreatment. The prior research reported that milling triggers mechanochemical effects, promoting the depolymerization and deacetylation of macromolecules in lignocellulosic biomass [20]. Mechanical fragmentation of lignocellulosic materials represents a critical step in the production of value-added by-products. The present findings confirm that ball milling offers a significant advantage, as it avoids the use of hazardous chemicals. This method presents strong environmental compatibility and holds considerable potential for the sustainable treatment and valorization of lignocellulosic residues.

### 3.2. Morphological Study

**Figure 2:**
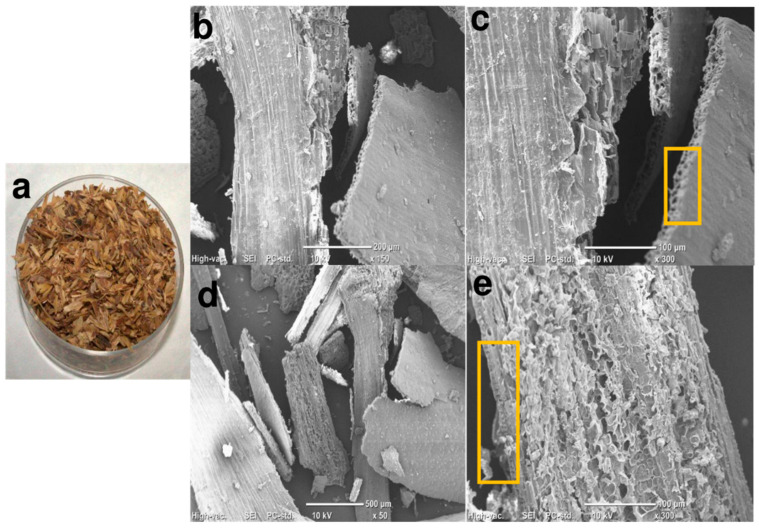
Images of (**a**) untreated brewer’s spent grain fibers (BSGFs) captured with a digital camera, (**b**) micrograph of the structure of untreated brewer’s spent grain fiber, (**c**) transverse section showing tubular conducts, (**d**,**e**) longitudinal sections of untreated BSGFs; obtained using SEM.

SEM analysis revealed the physical changes induced by ball milling. The extracted BSGFs displayed varied morphologies and sizes due to the treatment applied during extraction. These structures showed a smooth external surface (Figure 2b–d). In contrast, the internal surface appeared rough (Figure 2d). The cross-sectional view revealed an intermediate layer composed of spongy parenchyma surrounded by inner and outer epidermis (Figure 2c), consistent with structural features previously reported for plant fiber matrices [21].

**Figure 3:**
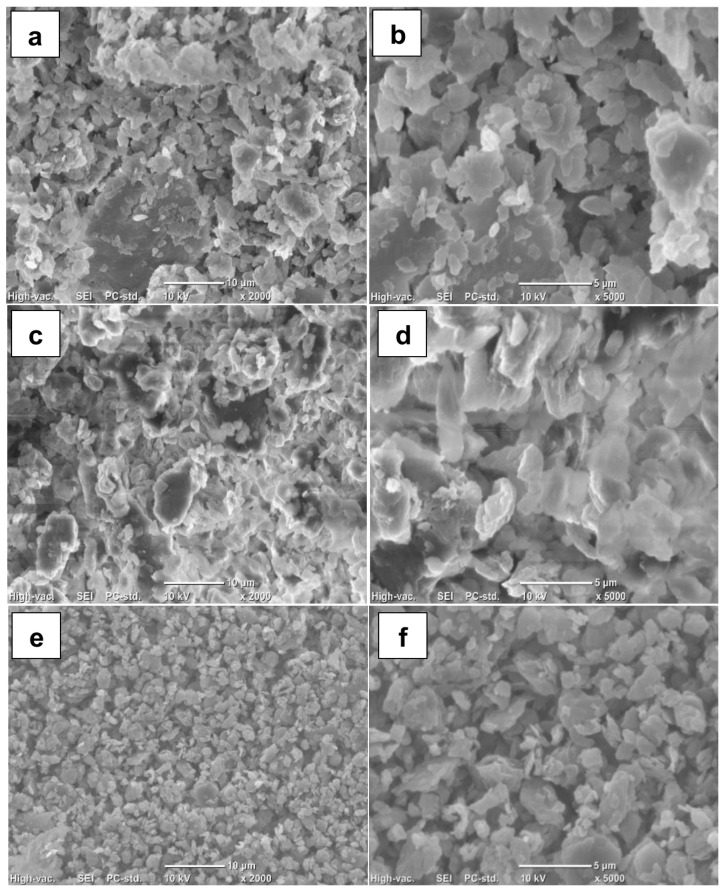
(**a**,**b**) Treatment after 120 min of milling at different magnifications, (**c,d**) treatment after 240 min of milling at different magnifications, (**e,f**) treatment after 360 min of milling at different magnifications.

Figure 3 shows the SEM images of BSGFs at different ball milling times. The images illustrate the transition from a defined surface structure to a less uniform morphology as treatment time increased. Impact, shear, and compression forces generated by collisions between the grinding media and the samples caused severe fragmentation of the original structure. This disruption produced porous, granular morphologies. The results highlight the effectiveness of ball milling in promoting defibrillation of the native fiber matrix, a desirable effect to produce value-added by-products. The high-impact milling process produced irregular, globular, and porous particles, consistent with the observations by Ji et al. [22], who reported similar morphologies when increasing milling time from 60 to 120 min. SEM images also showed that prolonged milling further reduced particle size, supporting the correlation between mechanical action intensity and the breakdown of lignocellulosic fiber networks.

According to the results, when milling time exceeded 120 min, the particles developed elliptical and quasi-circular shapes with rough, porous surfaces, as shown in Figure 3d. Khan et al. [11] also reported this morphology after applying milling treatment to a cellulose sample. Variations in the morphology of milled structures were observed [14,15], depending on milling conditions and treatment duration. After 360 min, the resulting microstructures appeared as amorphous microparticles. Du et al. [15] indicated that such amorphous particles facilitated the efficient conversion of cellulose into monomeric sugars during enzymatic hydrolysis. These results suggest that mechanical fractionation of lignocellulosic fibers represents a promising strategy for producing distinct sub-products suitable as feedstock in diverse applications.

As shown in Table 2, the ball milling process progressively reduced the particle size of the BSGF samples due to repeated collisions between the grinding media and the fibrous structures. This mechanical action caused the breakdown of larger fragments, resulting in the formation of smaller particles with greater surface exposure. After 120 min of treatment, the average particle size of sample T120 was 19.4 µm, suggesting that the fragmentation process reached the cellular level, which can enhance enzymatic accessibility in later applications. However, other studies have reported contrasting results depending on the biomass type and milling conditions applied [21]. In the case of the T360 sample, the average particle size increased slightly to 23.3 µm. These values closely match those reported by Gao et al. [23], who achieved a mean particle size of 23.60 µm in wheat straw subjected to high-energy milling for 1 h.

Baheti et al. [24] reported that agglomeration primarily occurs due to surface forces acting on particles in the dry state, especially when mechanical energy compacts finer structures. The milling pre-treatment effectively reduced the particle size, representing a suitable strategy for increasing the specific surface area of lignocellulosic substrates [25]. This increase enhances the accessibility of enzymes and reagents during bioconversion processes. Du et al. [15] previously demonstrated that reducing cellulose dimensions through milling was essential to accelerate hydrolysis and improve nanocellulose crystal production. Therefore, particle size reduction plays a key role in optimizing lignocellulosic valorization pathways.

### 3.3. Infrared Spectroscopy (FTIR)

**Figure 4:**
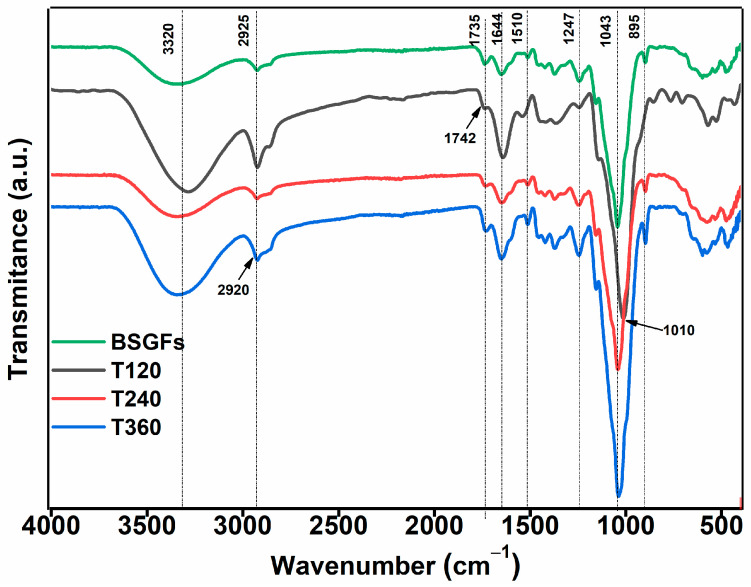
FTIR spectrum of untreated BSGFs and the different treatments at 120, 240, and 360 min.

Figure 4 presents the FTIR spectrum obtained for brewery spent grain fibers (BSGFs). The peak at 3320 cm^−1^ corresponds to O–H stretching vibrations, typically associated with the hydroxyl groups in cellulose [26,27]. The peak at 2923 cm^−1^ reflects C–H stretching in methyl and methylene groups, present in both cellulose and hemicellulose structures [28,29]. The peaks at 1735 cm^−1^ and 1644 cm^−1^ indicate C=O stretching of aldehyde groups in hemicelluloses and carboxyl groups in lignin, respectively [30]. The signal near 1510 cm^−1^ corresponds to aromatic ring deformations within lignin. Additionally, the peak at 1247 cm^−1^ is associated with aryl-alkyl ether bonds (C–O–C), a characteristic linkage in the lignin network [31].

A small peak at 1146 cm^−1^ indicates C–OH stretching vibrations associated with secondary alcohol groups in cellulose structures [32,33]. The band located at 1043 cm^−1^ corresponds to C–O stretching in ether linkages and hydroxyl groups, confirming the polysaccharide nature of the material [34]. Additionally, the peak observed at 895 cm^−1^ reflects C–O stretching in β-glycosidic bonds between glucose units, a structural feature characteristic of the cellulose backbone [35].

As shown in Figure 4, the ball milling treatment caused a noticeable broadening of the band corresponding to O–H groups. This effect reflects the disruption of intramolecular hydrogen bonding within cellulose and hemicellulose chains, a typical consequence of mechanical fragmentation. These results agree with previous observations reported by Zhang et al. [12], who described similar modifications in the hydrogen bonding pattern of milled lignocellulosic biomass. The band at 2923 cm^−1^ exhibited both a stretching vibration and a shift toward lower wavenumbers, resulting in a broader signal. This behavior indicates that ball milling influenced the molecular arrangement of cellulose, particularly affecting its crystalline structure.

In the milled samples, peaks shifted from their original positions to 1735 cm^−1^ and 1742 cm^−1^. These shifts reflect the alteration of ether bonds within hemicellulose, likely due to the mechanical forces involved in the process. Additionally, the peak at 1043 cm^−1^ moved to 1010 cm^−1^, which is associated with chain scission in hemicellulose. The peak at 895 cm^−1^ showed increased intensity, a pattern linked to the formation of amorphous cellulose, as also described by Lu et al. [36].

The spectral results indicate that ball milling effectively depolymerizes the lignin, disrupts the crystalline structure of cellulose, and significantly contributes to the fragmentation of hemicellulose chains present in bagasse fibers, thereby modifying the integrity of the lignocellulosic matrix at a molecular level.

### 3.4. Thermogravimetric Analysis (TGA)

**Figure 5:**
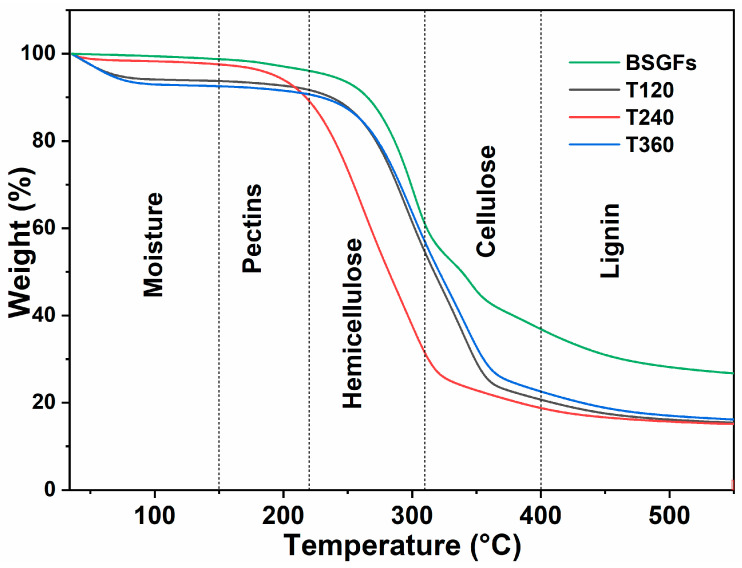
TGA curves of untreated brewery spent grain fiber and mechanically treated fibers at 120, 240, and 360 min.

Thermogravimetric analysis (TGA) was used to evaluate the thermal stability of BSGFs before and after mechanical treatment. The resulting thermal degradation profiles appear in Figure 5, illustrating changes in the decomposition behavior.

The graphs in Figure 5 represent the distinct phases of thermal degradation, characterized by the weight loss associated with the decomposition of the primary constituents of natural lignocellulosic fibers, including hemicellulose, cellulose, and lignin [34]. In general, both untreated and mechanically treated brewery spent grain fibers exhibited a three-stage degradation pattern. Before degradation began, a slight weight loss occurred between 35 and 150 °C, corresponding to the evaporation of moisture and the removal of waxy residues. The first main stage, observed between 226 and 309 °C, reflected the thermal breakdown of hemicellulose. The second stage, between 310 and 400 °C, was associated with the degradation of cellulose. Finally, the third stage, spanning from 410 to 600 °C, corresponded to the gradual decomposition of the lignin. These thermal behaviors align with the patterns reported for other natural fibers [26,28].

Among all treatments, sample T240 presented the highest weight loss within the hemicellulose degradation range (226–310 °C). This effect likely resulted from prolonged mechanical pre-treatment, which promoted extensive hemicellulose depolymerization into smaller oligosaccharides. These findings corroborate the results reported by Gao et al. [23], who observed similar thermal responses in pretreated biomass.

**Figure 6:**
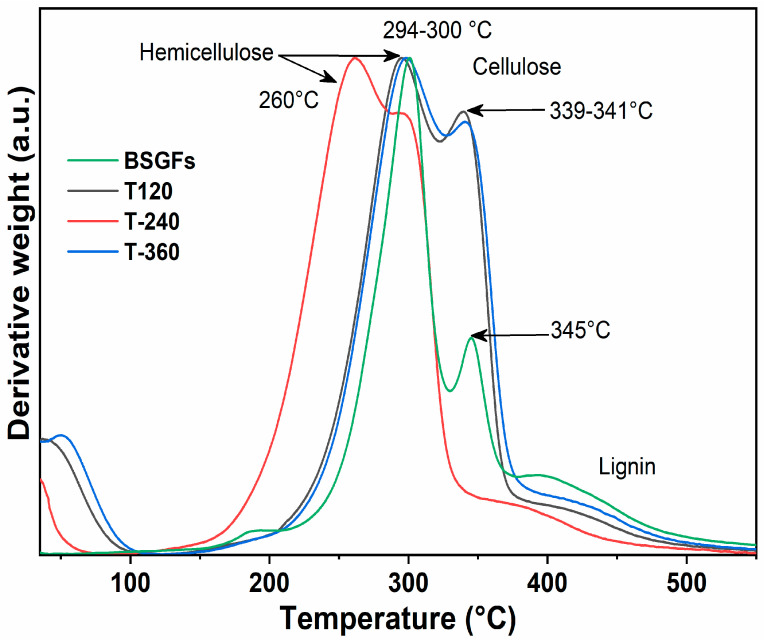
Graph of the derivative of weight loss for BSGFs and the different pre-treatments: T120, T240, and T360.

Figure 6 displays the first derivative thermogravimetric curves (DTG), which represent the rate of weight loss as a function of temperature for BSGFs and fibers subjected to mechanical treatment at different milling times.

Figure 6 illustrates that samples subjected to ball milling pre-treatment exhibited shifts in degradation temperatures, indicating a reduction in thermal stability compared to untreated natural fibers. For example, the T240 sample presented the lowest hemicellulose degradation temperature at 260 °C, while untreated BSGFs exhibited this transition at approximately 300 °C. This downward shift reflects the effect of mechanical action, which induces bond cleavage within the hemicellulose matrix and promotes depolymerization [12,37]. Consequently, structural destabilization leads to earlier thermal degradation.

A similar trend was observed for cellulose decomposition. The T120 sample showed a major decomposition peak at 339 °C, slightly lower than the 345 °C peak recorded for untreated BSGFs. This difference may result from the mechanical disruption of both amorphous and crystalline cellulose domains during milling. The reduction in thermal stability could be linked to a decrease in polymer chain length or to a partial transformation of crystalline cellulose into its amorphous counterpart. Amorphous cellulose generally exhibits lower thermal resistance. Reducing crystallinity is also advantageous for enhancing enzymatic hydrolysis efficiency in glucose production, as previously reported [38]. Ribeiro et al. [39] observed that prolonged ball milling reduced the crystallinity index of cellulose and simultaneously improved its catalytic conversion to sorbitol.

### 3.5. X-Ray Diffraction

**Figure 7:**
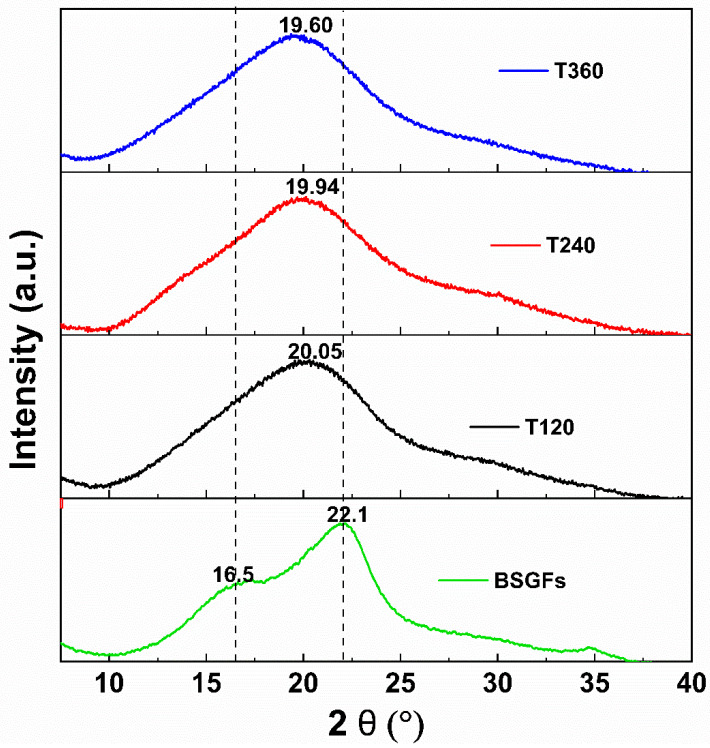
XRD analysis BSGFs and pre-treated fiber at 120, 240, and 360 min of milling.

Figure 7 shows the X-ray diffraction spectra of untreated BSGFs and ball-milled fibers.

Untreated brewer’s spent grain exhibited low-intensity diffraction peaks at 16.5° and 22.1°, which correspond to the crystalline and amorphous phases of cellulose, respectively [10,11]. In the ball-milled samples, the peak near 16.5° disappeared after 120 min of treatment, indicating the progressive loss of crystalline domains. Simultaneously, the peak at 22.1° became increasingly broader with longer milling times, suggesting the disruption of the crystalline lattice and the transition of cellulose into an amorphous structure. This peak broadening aligns with other studies and is considered a characteristic feature of amorphous cellulose [38].

The crystallinity index decreased significantly, from 34.21% in untreated BSGFs to 21.62% after 360 min of ball milling. These findings demonstrate that high-energy mechanical milling effectively disrupts the ordered arrangement of cellulose chains. This transformation results from mechanical forces transferred through repeated collisions between the milling media and the sample, causing severe structural rearrangements at the molecular level. Similar reductions in crystallinity have been reported in the literature. Ji et al. [22] observed that ball milling decreased the particle size of corn cob fibers and severely disrupted hydrogen bonding within cellulose chains. This disruption explains the reduced crystallinity index and confirms the efficacy of mechanical pretreatment in altering the structural integrity of lignocellulosic materials.

The results confirmed that ball milling disrupted the crystalline structure of cellulose. This reduction in crystallinity offers advantages for specific applications, as it improves enzymatic hydrolysis efficiency, increases the overall yield of fermentable sugars, and enhances the biodigestibility of lignocellulosic biomass during bioconversion processes.

## 4. Conclusions

Mechanical milling substantially altered the chemical composition of brewer’s spent grain, increasing the cellulose content to a maximum of 45.17% and reducing the lignin content to 26.83%. These compositional changes resulted from the fragmentation induced by ball milling. The SEM analysis revealed morphological transformations, including particle size reduction and the formation of porous, spherical structures. FTIR spectroscopy identified modifications in cellulose and hemicellulose bonding patterns, reflecting hydrogen bond disruption and partial depolymerization. XRD analysis confirmed a reduction in crystallinity index, indicating a shift from crystalline to amorphous cellulose. TGA results showed decreased thermal degradation temperatures for hemicellulose and cellulose, consistent with molecular fragmentation. These findings highlight the potential of mechanical pretreatment to enhance the value of agro-industrial waste. The processed material exhibits promising characteristics for use in sustainable industrial applications.

## Figures and Tables

**Table 1:** Chemical composition of brewer’s spent grain, brewer’s spent grain fibers, and treatments at 120, 240, and 360 min of milling pre-treatment.

Fiber	% Cellulose	Hemicellulose Content	% Lignin	% Soluble Extractible Compounds	Reference
Brewer’s spent grain	27.61	32.62	28.89	8.6	Current Study
Brewer’s spent grain	26.80	37.17	17.13	-	[18]
Brewer’s spent grain	24.5	23.8	15.8	-	[19]
Brewer’s spent grain fibers (BSGFs)	36.78	41.82	27.82	15.58	Current Study
Microfiber (T120)	41.97	23.38	30.98	14.32	Current Study
Microfiber (T240)	34.93	41.26	29.70	12.13	Current Study
Microfiber (T360)	45.17	27.86	26.83	13.96	Current Study

**Table 2:** Particle size distribution of different samples obtained after 120, 240, and 360 min of milling.

	Milling Time (min)		
Particle Size (µm)	T120	T240	T360
Mean	19.4 ± 0.74	26.9 ± 1.68	23.3 ± 0.81
Median	14.9 ± 0.35	21.5 ± 1.36	17.6 ± 0.71
Mode	19.2 ± 1.01	27.9 ± 1.47	25.4 ± 1.34

Data are given as average values ± the standard deviation.

## Data Availability

The original contributions presented in the study are included in the article, further inquiries can be directed to the corresponding author.
